# The complete chloroplast genome sequence of *Trapa incisa* Sieb. & Zucc. (Lythraceae)

**DOI:** 10.1080/23802359.2021.1930601

**Published:** 2021-06-02

**Authors:** Wuchao Wang, Xiangrong Fan, Xiuling Li, Yuanyuan Chen

**Affiliations:** a Hubei Key Laboratory of Wetland Evolution & Ecological Restoration, Wuhan Botanical Garden, Chinese Academy of Sciences, Wuhan, P. R. China; b College of Life Science, University of Chinese Academy of Sciences, Beijing, P. R. China; c College of Science, Tibet University, Lhasa, P. R. China; d Research Center for Ecology, and Environment of Qinghai-Tibetan Plateau, Tibet University, Lhasa, P. R. China; e College of Life Science, Linyi University, Linyi, P. R. China; f Key Laboratory of Aquatic Botany and Watershed Ecology, Wuhan Botanical Garden, Center of Plant Ecology, Core Botanical Gardens, Chinese Academy of Sciences, Wuhan, P. R. China

**Keywords:** *Trapa incisa*, complete chloroplast genome, Lythraceae, phylogeny

## Abstract

*Trapa* L., an annual floating-leaved herb, is widely distributed in the old world and has important edible and medicinal values. However, the taxonomy and phylogeny of *Trapa* are unclear. Here, we reported the complete chloroplast genome of a wild species with small nuts, *T. incisa*. The complete chloroplast genome size of *T. incisa* was 155, 453 bp, consisting of two inverted repeat (IR) regions (24, 388 bp), one large single copy (LSC) region (88, 398 bp) and one small single copy (SSC) region (18, 279 bp). A total of 129 genes were annotated, including 83 protein-coding genes, 38 tRNA genes and 8 rRNA genes. Among them, 19 genes were duplicated (6 protein-coding genes, 9 tRNA genes and 4 rRNA genes). The phylogenomic analysis suggested a close relationship between *T. incisa* and *T. maximowiczii*.

Water chestnut *Trapa* L. (Lythraceae) is an annual floating-leaved aquatic herb native to the temperate to subtropical regions of Africa, Asia, and Europe (Chen et al. 2007). Besides the important ecological values, the *Trapa* plants have been commercially cultivated as edible fruits in India, China and Italy (Suriyagoda et al. 2007). However, because of the various morphological traits and shortage of effective identification methods, the taxonomy and phylogeny of the genus are still unclear (Kim et al. 2010; Li et al. 2017). Molecular information is urgently needed to improve the situation of the genus. Previous studies showed that the nut size offered the best diagnostic criteria for the classification of *Trapa* species (Xiong et al. 1990; Fan et al. 2016). *Trapa incisa* is a typical species with small nut size. In this study, the chloroplast genome sequence of *T. incisa* was released, and the phylogenetic relationship was reconstructed within Lythraceae. The basic genetic information is helpful to species identification and systematic relationships construction within *Trapa*.

An individual of *T. incisa* was collected from the Wuhan Botanical Garden, Chinese Academy of Sciences, Hubei, China (114.613°E; 30.543°N). The voucher specimen was deposited at the Herbarium of Wuhan Botanical Garden (HIB: Yuanyuan Chen, yychen@wbgcas.cn) under the voucher number yychen20180066. Genome DNA was isolated from 0.5 g fresh leaves using the modified CTAB method (Doyle and Doyle 1987). The purified DNA was used to build a sequencing library with the Illumina NovaSeq 6000 platform. Finally, a total of 5.31 G raw data was obtained for further analysis. The complete chloroplast genome was assembled by GetOrgnelle v1.71 (Jin et al. 2020). The resultant genome was annotated by the genome annotator GeSeq (Tillich et al. 2017) with *T. bicornis* and *T. maximowiczii* as references; and the results were manually adjusted by Geneious (Kearse et al. 2012). The complete annotation chloroplast genome of *T. incisa* was deposited in GenBank with an accession number of MW543307.

The complete chloroplast genome length for *T. incisa* was 155, 453 bp with the quadripartite structure, including two inverted repeat (IR) regions (24, 388 bp), one large single copy (LSC) region (88, 398 bp) and one small single copy (SSC) region (18, 279 bp). The overall GC content was 36.4%. A total of 129 genes were annotated, consisting of 83 protein-coding genes, 38 tRNA genes and 8 rRNA genes. Among them, 19 genes were duplicated, including 6 protein-coding genes, 9 tRNA genes and 4 rRNA genes.

## Phylogenetic analyses

**Figure 1.**
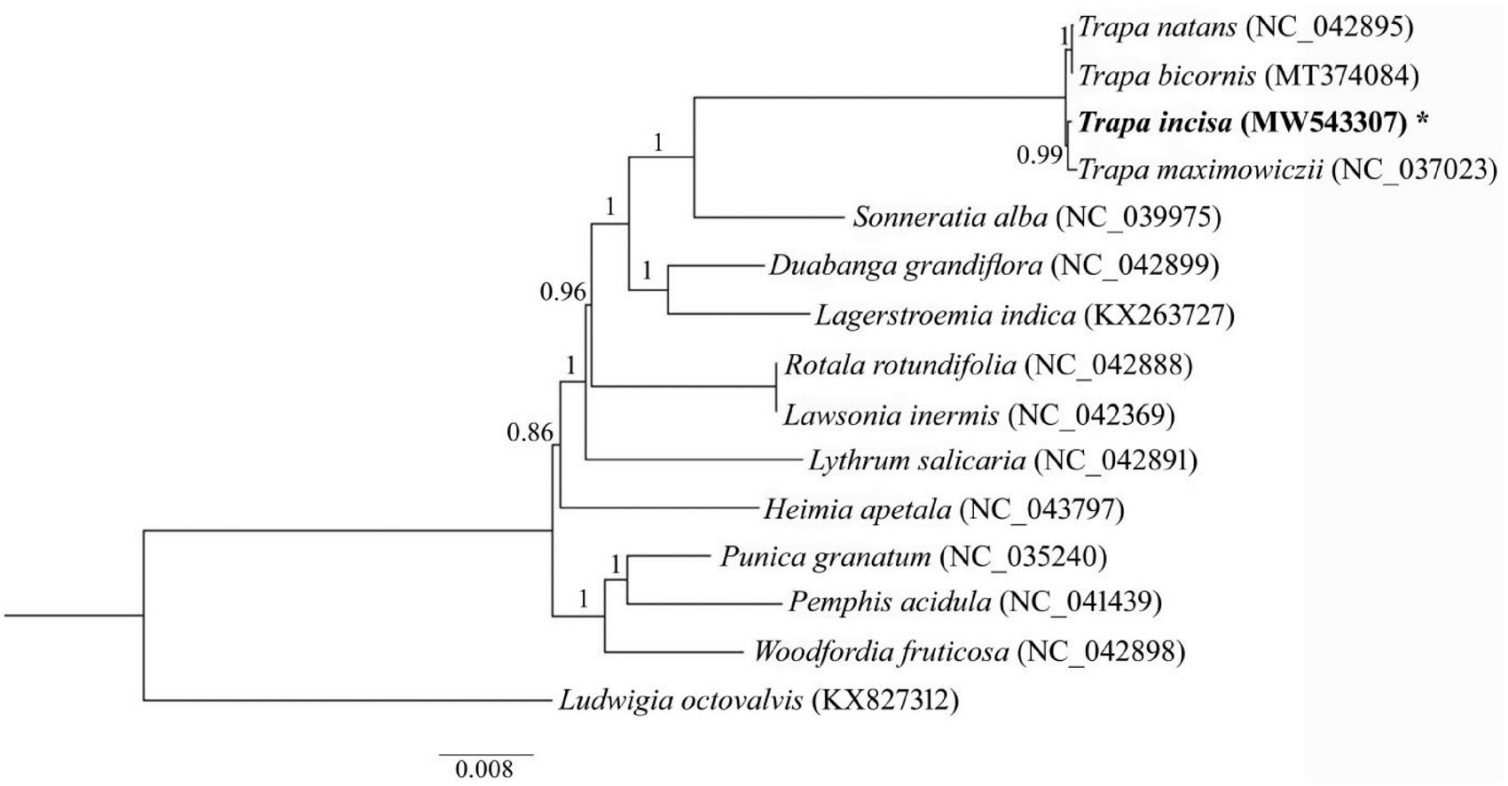
Phylogenetic tree using maximum-likelihood (ML) based on plastomes of 14 Lythraceae species with *Ludwigia octovalvis* as an outgroup. Numbers near the nodes represent ML bootstrap values.

A maximum likelihood (ML) phylogenetic tree was constructed based on the 14 published complete plastomes of Lythraceae, with *Ludwigia octovalvis* (NC031385) as an out-group. The genome sequences were initially merged by BioEdit (Hall 1999), then aligned using MAFFT (Katoh et al. 2019). The ML tree was computed by PhyML v.3.0 (Stéphane et al. 2010) under the best model (TVM + G + I) and evaluated by Jmodeltest (Darriba et al. 2012). The phylogenetic tree strongly supported a close relationship between *T. incisa* and *T. maximowiczii,* which all have small seeds (Figure 1). Additionally, *Sonneratia* was closely related to *Trapa* within the family Lythraceae, which was also suggested by previous studies (Graham et al. 2005).

## Data Availability

The genome sequence data that support the findings of this study are openly available in GenBank of NCBI at (https://www.ncbi.nlm.nih.gov/nuccore/MW543307.1/) under the accession no. MW543307. The associated BioProject, SRA, and Bio-Sample numbers are PRJNA726367, SRX10752768, and SAMN18928742, respectively.
